# Early identification of patients at high risk of group A streptococcus-associated necrotizing skin and soft tissue infections: a retrospective cohort study

**DOI:** 10.1186/s13054-019-2708-y

**Published:** 2019-12-21

**Authors:** Tomas Urbina, Camille Hua, Paul-Louis Woerther, Armand Mekontso Dessap, Olivier Chosidow, Nicolas de Prost

**Affiliations:** 1 Service de Réanimation Médicale, Hôpitaux Universitaires Henri Mondor, Assistance Publique – Hôpitaux de Paris (AP-HP), Créteil, France; 2 Service de Dermatologie, Hôpitaux Universitaires Henri Mondor, Assistance Publique – Hôpitaux de Paris (AP-HP), Créteil, France; 3 Université Paris-Est Créteil Val de Marne (UPEC), Créteil, France; 4 Laboratoire de Bactériologie-Hygiène, Hôpitaux Universitaires Henri Mondor, Assistance Publique – Hôpitaux de Paris (AP-HP), Créteil, France; 5 Équipe EA 7380 Dynamyc, Unité de Formation et de Recherche (UFR) de Médecine - site Créteil, Université Paris-Est Créteil Val-de-Marne, Créteil, France; 6 Groupe de Recherche Clinique CARMAS, Université Paris Est-Créteil, Créteil, France

**Keywords:** Necrotizing soft tissue infection, Group A streptococcus, Toxin, Intravenous immunoglobulins, Clindamycin

Dear Editor,

Necrotizing soft tissue infections (NSTIs) are a heterogeneous group of devastating diseases involving a wide variety of microorganisms and affecting different body areas. The need for individualized treatment strategies has been recently put forward in a prospective cohort study of 402 patients in which group A streptococcus (GAS) infections were associated with more frequent septic shock [1]. Early identification of patients with GAS-related NSTIs could prompt initiation of targeted interventions, including clindamycin and intravenous immunoglobulins (IVIg). These drugs might be associated with beneficial anti-toxinic properties, but the level of evidence supporting them remains low (clindamycin) or highly controversial (IVIg) [2, 3]. The only randomized clinical trial evaluating the effect of IVIg specifically in patients with NSTI could not demonstrate a benefit on a composite outcome of death and quality-of-life evaluation at 6 months [4]. As previously commented [5], only 15% (*n* = 13/87) of included patients eventually had a microbiologically proven GAS NSTI. This was a major limitation and early identification of patients with a high probability of GAS-associated NSTIs would thus be crucial for further studies evaluating similar interventions.

A secondary analysis of a retrospective cohort including 224 patients admitted to our center for NSTI between 2006 and 2017 was conducted [6]. In accordance with the most recent guidelines, only patients with surgically confirmed NSTI were included (i.e., macroscopic appearance of tissues during operation as swollen, dull gray with a thin, brownish exudate with or without necrosis). Admission characteristics and microbiological documentation based on surgical samples, blood cultures, or subcutaneous puncture were recorded. We compared patients with a documented GAS infection to other patients regarding admission characteristics. A multivariable logistic regression model was used to identify admission characteristics associated with a subsequent GAS documentation.

Among 224 patients, 60 (27%) had a GAS infection, which was monomicrobial in 39 (17%) cases. Overall, 134 (59.8%) patients were admitted to the intensive care unit during their stay, of whom 113 during the first 24 h. Ninety-one (41%) patients presented with shock (i.e., required vasopressors), and 89 (40%) required mechanical ventilation. Sixty days after admission, 51 (23%) patients had died, including 10 (17%) with GAS, and 41 (25%) with non-GAS infections (*p* = 0.255, Mann-Whitney test). Admission characteristics associated with GAS infections by univariable analysis were non-steroidal anti-inflammatory drug treatment before admission and leukocytosis as a continuous variable. Those inversely associated with GAS infections were immunodeficiency, the nosocomial onset of infection, and an abdominoperineal location (Table 1). After multivariable analysis, only immunodeficiency (adjusted odds ratio (aOR) = 0.29 [0.10–0.74], *p* = 0.015) and an abdominoperineal location (aOR = 0.06 [0.00–0.30], *p* = 0.007) remained associated with the absence of GAS infection (Table 1). A sensitivity analysis using “monomicrobial GAS NSTI” as the dependent variable yielded similar results, except for younger age that remained in the model after adjustment (data not shown). Immunodeficiency (*n* = 58) and an abdominoperineal location (*n* = 38) had respective positive predictive values for the absence of a GAS infection (both mono- or polymicrobial) of 90% [79–96] and 97% [86–100] (Fig. 1).

**Table 1:** Admission characteristics associated with group A streptococcal documentation

	Available data	Overall (*n* = 224)	GAS^a^ (*n* = 60)	Others (*n* = 164)	*p* (univariate)^b^	Adjusted OR^c^	*p* (multivariate)^c^
Demographical data							
Age, years, median (IQR)	224	64.00 [53.00–74.25]	60.00 [50.00–72.00]	65.00 [55.50–75.00]	0.083		
Male gender, *n* (%)	224	127 (56.7)	31 (51.7)	96 (58.5)	0.443		
Comorbidities, *n* (%)							
Diabetes mellitus	224	83 (37.1)	18 (30.0)	65 (39.6)	0.244		
Immunodeficiency	224	58 (25.9)	6 (10.0)	52 (31.7)	0.002	0.29 [0.10–0.74]	0.015
HIV infection	224	2 (0.9)	0 (0.0)	2 (1.2)	0.954		
Cancer	224	21 (9.4)	0 (0.0)	21 (12.8)	0.008		
Corticosteroids	224	36 (16.1)	6 (10.0)	30 (18.3)	0.197		
Obliterating arteritis of the lower limbs	224	24 (10.7)	5 (8.3)	19 (11.6)	0.651		
Liver cirrhosis	224	9 (4.0)	0 (0.0)	9 (5.5)	0.142		
Chronic kidney disease	224	25 (11.2)	4 (6.7)	21 (12.8)	0.293		
Chronic alcohol consumption	224	27 (12.1)	5 (8.3)	22 (13.4)	0.422		
Obesity	224	57 (25.4)	13 (21.7)	44 (26.8)	0.54		
Prior to admission							
Time from first symptom, days, median (IQR)	224	5.00 [2.00–9.75]	5.00 [2.00–7.25]	5.00 [2.00–10.00]	0.599		
Antibiotic treatment, *n* (%)	221	137 (61.2)	30 (50.8)	107 (66.0)	0.057		
NSAID use, *n* (%)	222	46 (20.5)	19 (31.7)	27 (16.7)	0.024	–	0.122
Presentation upon admission							
Nosocomial infection, *n* (%)	222	45 (20.1)	4 (6.7)	41 (25.3)	0.004	–	0.197
Abdominoperineal location, *n* (%)	223	38 (17.0)	1 (1.7)	37 (22.7)	< 0.001	0.06 [0.00–0.30]	0.007
Shock, *n* (%)	220	91 (40.6)	21 (35.6)	70 (43.5)	0.369		
Creatininemia, μmol/L, median [IQR]	210	112.50 [69.00–171.25]	123.00 [71.25–187.25]	109.50 [67.25–167.75]	0.571		
Uremia, mmol/L, median [IQR]	207	9.80 [5.25–19.00]	10.25 [5.45–18.02]	9.80 [5.20–19.10]	0.966		
Plasma bicarbonate, mmol/L, median [IQR]	193	22.90 [19.70–26.80]	22.70 [20.10–26.00]	23.00 [19.50–27.05]	0.943		
Blood leucocytes 10^3^/mm3, median [IQR]	219	14.40 [9.50–21.60]	17.20 [12.35, 22.50]	13.60 [9.00–21.00]	0.016	–	0.067
Platelets 10^3^/mm3, median [IQR]	189	217.00 [153.00–329.00]	223.50 [181.25–312.50]	207.00 [144.00–344.00]	0.45		
Hemoglobinemia, g/dL, median [IQR]	215	10.70 [9.45–12.15]	11.05 [10.15–12.50]	10.60 [9.35–12.10]	0.171		
Arterial lactate-mmol/L median [IQR]	146	2.00 [1.30–3.48]	2.10 [1.50–3.60]	2.00 [1.20–3.40]	0.677		

Analysis among 224 patients admitted for necrotizing soft tissue infection. ^a^Group A streptococcal infection. ^b^*p* values for univariate comparison of documented group A streptococcal infection vs others; Chi-squared test or Fisher’s exact test were used for categorical data according to sample size, Mann-Whitney’s test was used for continuous variables due to non-parametrical distribution. ^c^*p* values and adjusted ORs from a logistic regression model assessing the relationship between admission characteristics and group A streptococcal documentation. The model included all variables with a *p* value < 0.05 in univariate analysis. Analysis regarding 213 patients (11 patients excluded for missing data on one of the variables of the model. Immunodeficiency encompassed active cancer, chemotherapy within the last 3 months, previous HIV infection whatever the AIDS status, the CD4 lymphocytes counts or the viral load, any immunosuppressive drugs including chronic systemic steroid treatment (whatever the dose but for at least 3 months). *HIV* human immunodeficiency virus, *NSAID* non-steroidal anti-inflammatory drug

**Fig. 1:**
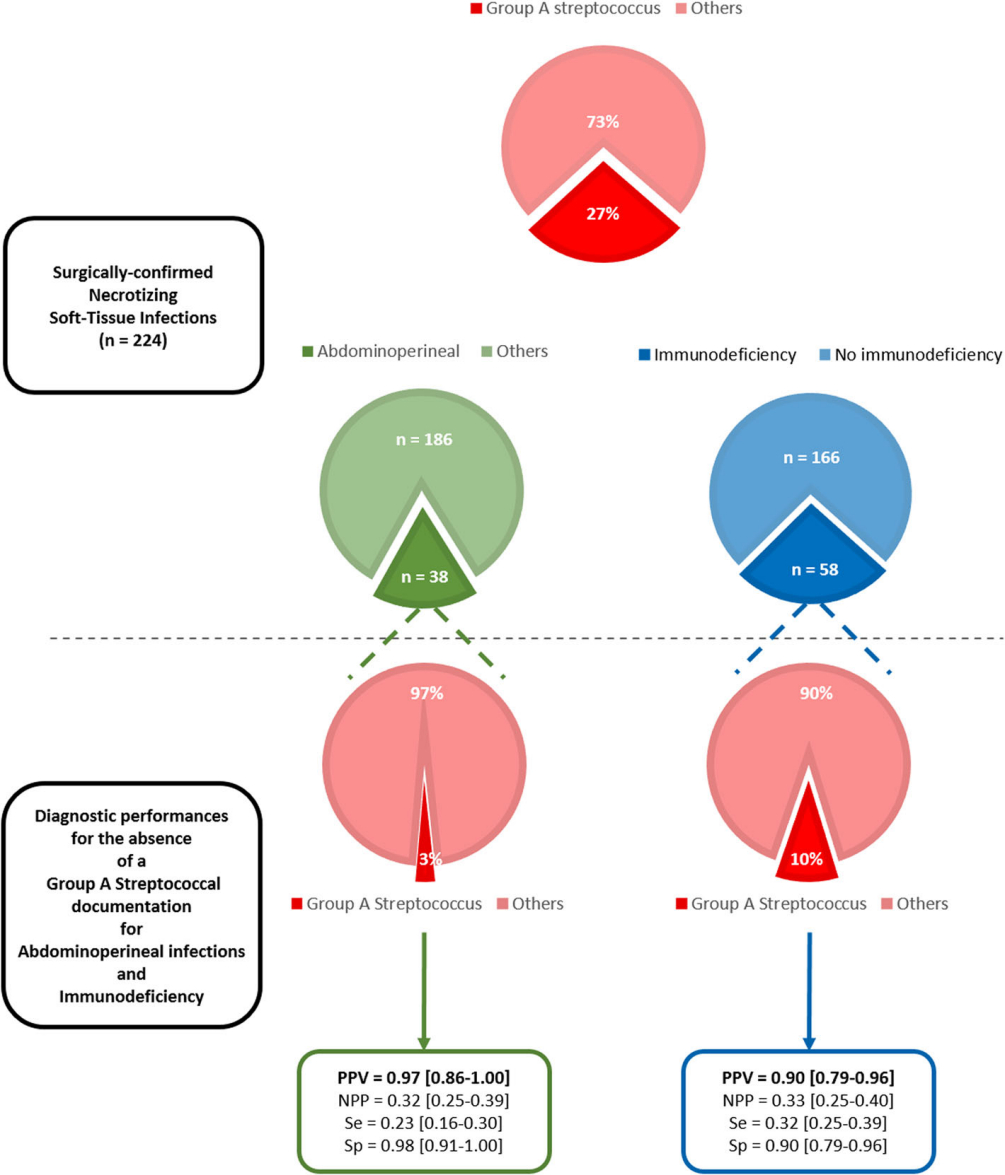
Diagnostic performances of abdominoperineal location and immunodeficiency for predicting absence of group A streptococcal documentation. The three top pie charts represent the proportions of group A streptococcal documentation, abdominoperineal infections and immunodeficiency in the whole 224-patient population of surgically confirmed necrotizing soft tissue infections. The two bottom pie charts represent the proportion of group A streptococcal documentation in the subgroup of patients with abdominoperineal infections (bottom left chart) or in immunocompromised patients (bottom right chart). Diagnostic performances of an abdominoperineal location of infection and of immunodeficiency for predicting the absence of group A streptococcal documentation were calculated using a contingency table approach. Immunodeficiency encompassed active cancer, chemotherapy within the last 3 months, previous HIV infection whatever the AIDS status, the CD4 lymphocytes counts or the viral load, any immunosuppressive drugs including chronic systemic steroid treatment (whatever the dose but for at least 3 months). PPV, positive predictive value; NPP, negative predictive value; Se, sensitivity; Sp, specificity

In conclusion, we retrospectively identified two simple and available upon admission clinical predictors of GAS documentation among a large cohort of surgically proven NSTIs. Our results show that NSTI patients with pre-existing immunodeficiency or an abdominal infection have a low probability of GAS infection and might thus not be suitable for inclusion in a trial assessing the effect of GAS-specific interventions. Such findings need to be assessed in a validation cohort in order to reinforce their generalizability. Improving identification upon admission of a subgroup of patients with a higher prevalence of GAS infection might help design future prospective trials aimed at assessing personalized treatment strategies [2].

## Data Availability

The dataset used during the current study is available from the corresponding author upon reasonable request.

## Abbreviations

GAS: Group A streptococcus
IVIG: Intravenous immunoglobulins
NSTI: Necrotizing soft tissue infection
OR: Odds ratio
PPV: Positive predictive value
NPP: Negative predictive value
Se: Sensitivity
Sp: Specificity
